# Osteoarthritis accelerates and exacerbates Alzheimer's disease pathology in mice

**DOI:** 10.1186/1742-2094-8-112

**Published:** 2011-09-07

**Authors:** Stephanos Kyrkanides, Ross H Tallents, Jen-nie H Miller, Mallory E Olschowka, Renee Johnson, Meixiang Yang, John A Olschowka, Sabine M Brouxhon, M Kerry O'Banion

**Affiliations:** 1 Department of Children's Dentistry, Stony Brook University Health Science Center, Stony Brook NY 11794, USA; 2 Department of Emergency Medicine, Stony Brook University Health Science Center, Stony Brook NY 11794, USA; 3 Department of Oral Biology & Pathology, Stony Brook University Health Science Center, Stony Brook NY 11794, USA; 4 Eastman Institute for Oral Health, University of Rochester Medical Center, Rochester NY 14620, USA; 5 Neurobiology & Anatomy, School of Medicine & Dentistry, University of Rochester, Rochester NY 14642, USA

## Abstract

**Background:**

The purpose of this study was to investigate whether localized peripheral inflammation, such as osteoarthritis, contributes to neuroinflammation and neurodegenerative disease *in vivo*.

**Methods:**

We employed the inducible Col1-IL1β^XAT ^mouse model of osteoarthritis, in which induction of osteoarthritis in the knees and temporomandibular joints resulted in astrocyte and microglial activation in the brain, accompanied by upregulation of inflammation-related gene expression. The biological significance of the link between peripheral and brain inflammation was explored in the APP/PS1 mouse model of Alzheimer's disease (AD) whereby osteoarthritis resulted in neuroinflammation as well as exacerbation and acceleration of AD pathology.

**Results:**

Induction of osteoarthritis exacerbated and accelerated the development of neuroinflammation, as assessed by glial cell activation and quantification of inflammation-related mRNAs, as well as Aβ pathology, assessed by the number and size of amyloid plaques, in the APP/PS1; Col1-IL1β^XAT ^compound transgenic mouse.

**Conclusion:**

This work supports a model by which peripheral inflammation triggers the development of neuroinflammation and subsequently the induction of AD pathology. Better understanding of the link between peripheral localized inflammation, whether in the form of osteoarthritis, atherosclerosis or other conditions, and brain inflammation, may prove critical to our understanding of the pathophysiology of disorders such as Alzheimer's, Parkinson's and other neurodegenerative diseases.

## Background

Systemic (peripheral) inflammation may be associated with increased risk for Alzheimer's Disease (AD) pathology. In particular, a number of investigators have reported associations between serum levels of pro-inflammatory cytokines and other markers, including interleukin (IL)-1β, IL-6, tumor necrosis factor (TNF)α, C-reactive protein and α1-antichymotrypsin, with increased risk for dementia and AD [1-6]. Increased risk for AD was also observed in people homozygous for allele 2 of IL-1β (+3953), a variant previously associated with increased IL-1β secretion *in vitro *[7,8].

In animal models of neurodegeneration, experimentally-induced acute systemic inflammation led to the release of proinflammatory factors in the central nervous system that exacerbated neurodegeneration [9,10]. In another study, repeated intraperitoneal lipopolysaccharide (LPS) injection in wild type male mice resulted in accumulation of Aβ1-42 in the hippocampus and cerebral cortex [11]. In contrast, LPS administration to AD mouse models has given mixed results, with some investigators reporting exacerbation [12-14] and others improvement of pathology due to an inflammation-induced phagocytic activity [15-17]. To this end, over-expression of inflammatory cytokines in the brain of AD mouse models also resulted in alleviation of AD pathology [18], including our own where sustained expression of interleukin-1β (IL-1β) in mouse hippocampus promoted plaque clearance in the APP/PS1 double transgenic mouse model [19].

But how is peripheral inflammation linked to AD pathology? Osteoarthritis (OA) in particular manifests as a slowly progressing debilitating disease that affects one or more joints of the body. Clinical symptoms include pain, swelling, joint enlargement and decreased range of joint motion. Substantial evidence confirms the role of proinflammatory cytokines, including IL-1β, as mediators in disease development [20-22]. To explore whether osteoarthritis contributes to the development of neuroinflammation and possibly AD pathology, we employed somatic mosaic expression of IL-1β in the knees and temporomandibular joints of the Col-IL1β^XAT ^transgenic mouse model of osteoarthritis [23-25]. We report that localized induction of osteoarthritis in the young adult APP/PS1 mouse model of AD leads to glial activation as well as acceleration and exacerbation of AD plaque pathology. A link between peripheral and brain inflammation may prove critical to our understanding of neurodegenerative disorders and treatments thereof.

## Methods

### Animal studies

All experimental protocols involving animals were reviewed and approved by the University Committee on Animal Resources (IACUC). Employing a somatic mosaic analysis approach, we induced osteoarthritis in knees and temporomandibular joints (TMJs) of the Col1-IL1β^XAT ^mouse model [23-25]. Under anesthesia (ketamine 40 mg/kg intraperitoneally), 2 month old Col1-IL1β^XAT ^transgenic mice received bilateral intra-articular injections of FIV(Cre) in both knees and temporomandibular joints (10 μL solution containing a total of 10^6 ^infectious particles per joint) as previously described. In addition, Col1-IL1β^XAT ^mice that received equal dose/volume of FIV(gfp) or saline served as controls. Two or 6 months after viral transduction, the mice were deeply anesthetized (pentobarbital 100 mg/Kg intraperitoneally) and decapitated. A total of 32 mice was employed in this study: 13 Col1-IL1β^XAT ^mice injected with FIV(Cre), 13 mice injected with FIV(gfp) and 6 mice injected with saline intra-articularly. Their brains were harvested and split sagitally in two halves: one half was fixed by immersion in 10% formalin for immunohistochemical analysis and the other half was immersed into Trizol reagent (Invitrogen) for RNA extraction. In addition, blood serum was collected for assessment of human IL-1β and murine IL-6 levels by ELISA (R&D Systems, Minneapolis MN).

The biological significance of arthritis-induced neuroinflammation was evaluated in the APP/PS1 mouse model of Alzheimer's disease [26]. To this end, APP/PS1; Col1-IL1β^XAT ^compound transgenic mice were generated on the C57/BL6 background strain by crossing Col1-IL1β^XAT ^transgenic mice into the APP/PS1 (B6C3-Tg(APPswe, PSEN1dE9)85Dbo/J) mouse model obtained from The Jackson Laboratories (stock 4462; Bar Harbor, ME). Osteoarthritis was induced in the knees and TMJs of 2 month old APP/PS1; Col1-IL1β^XAT ^mice by FIV(Cre) injection (10 μL solution containing a total of 10^6 ^infectious particles per joint) under anesthesia. The mice were sacrificed 2 and 6 months following viral transduction of the knees and TMJs, at the age of 4 and 8 months, respectively. A total of 38 mice was employed in this study, including 18 experimental (APP/PS1; Col1-IL1β^XAT^) mice with arthritis and 20 (APP/PS1) control mice: The male:female ratio was 1:1.

### Histology

Brain histology sections were cut on a freezing microtome into 18 μm thick sections, which were collected on Superfrost^® ^glass slides. Immunohistochemical analysis for glial fibrillary acidic protein (GFAP) and class II major histocompatibility complex (MHC-II) was performed using a rabbit anti-GFAP (human) polyclonal antibody (1:1,000 dilution; Dako USA, Carpinteria, CA), and a rat anti-MHC-II (mouse) antibody (1:500 dilution; Bachem, Torrance CA; clone ER-TR3), respectively. Aβ plaques were identified by immunohistochemistry employing a mouse anti- β-amyloid (rodent) monoclonal antibody (1:400 dilution; SIGNET, El Monte, CA; clone 6E10). For Aβ staining, brain sections were treated in 90% formic acetate aqueous solution for 5 minutes prior to immunohistochemistry. Primary antibodies were coupled with appropriate secondary antibodies: goat anti-rabbit IgG biotin-conjugated and goat anti-rat IgG biotin-conjugated antibodies, respectively (Jackson Immunoresearch, West Grove PA). Visualization was performed utilizing DAB (3,3-diaminobenzidine)-nickel as chromagen. Slides were dehydrated through multiple ethanol solutions, cleared through xylene and cover-slipped using DPX permanent mounting medium (Fluka, Neu-Ulm, Switzerland). Tissue sections were examined under a BX51 Olympus light microscope and color microphotographic images were captured. The total numbers of GFAP^+^, MHC-II^+ ^cells were counted in 10 random microscopic fields (40×) and cell counts were expressed as averages (± standard errors of mean) for each antigen. The number of Aβ plaques were counted on histology sections, divided into an anterior, middle and posterior third of the brain and expressed as averages (± standard errors of mean) for each animal group. Each brain was halved midsagittally and sectioned on a cryostat into 20 μm thick coronal sections collected sequentially onto 12 Permafrost^® ^glass slides, such that a total of 24 sections were on each glass slide and each section represented an area of the brain that was 240 μm apart from each neighboring section. The first 8 sections on each glass slide represented the anterior portion of the brain, the next 8 sections the middle and the last 8 sections represented the posterior third of the brain in our cell counting.

The knees and temporomandibular joints were also harvested, defleshed and decalcified by immersion in an EDTA solution for 7-14 days in 4°C under constant agitation. The joints were then processed on a RHS-1 microwave tissue processor, after which the samples were embedded in paraffin, cut on a microtome as 3 μm thick sections and collected on glass slides. Joint histopathology was evaluated in sections stained by Alcian blue-orange G histochemistry using a scale 0-5 previously described [23,24]. Articular cloning was assessed by microscopy and counted as 2 or more chondrocytes present in a single lacuna in each joint section [23]. Antibodies used in these experiments include a rabbit anti-human mature IL-1β (1:100; Abcam, Cambridge MA), and rabbit anti-β-galactosidase (bacterial) (1:1,000; Sigma; St. Louis, MO). Cre recombinase expression was assessed with an antibody raised against its V5 fusion epitope (1:500; rat anti-V5; Invitrogen).

### RT-PCR

Quantification of mRNA levels was accomplished using an iCycler (Bio-Rad) and real time qRT-PCR with Taqman probes constructed with FAM (fluorescent marker) and Blackhole I quencher (Biosearch Technologies, Novato CA) as previously described [23]. PCR reactions were performed in a volume of 25 μl and contained iQ Supermix (Bio-Rad, Hercules CA), 0.625 U Taq, 0.8 mM dNTP, 3 mM Mg^2+^, 0.2-0.6 μM concentrations of each primer, 10-100 nM probe and 1 μl of cDNA sample. To correct for variations in starting RNA values, the level of ribosomal 18S RNA or GAPDH RNA was determined for all samples and used to normalize all subsequent RNA determinations. Normalized threshold cycle (Ct) values were then transformed, using the function- expression = (1+ e) Ct, in order to determine the relative differences in transcript expression. Using this method, transcript levels for IL-1β, TNFα, GFAP and MHC-II were measured.

### Behavioral Analyses

Grooming behavior was evaluated by adapting a method previously described [23]. In brief, mice were placed in a custom-made cage (12"x12"x12") with 4 mirrored walls. The cage lacked a roof so that the mice could be observed and recorded. Each mouse was transferred into the aforementioned observation chamber containing bedding from its original cage and was allowed a 30 min habituation period to minimize stress. Behaviors were recorded on a video-tape for a period of 60 minutes using a Sony digital recorder (Digital Handycam/Digital 8) with a Cokin macro digital lens (mode C043) added for image enlargement. The mouse was then returned to its original cage. Grooming was measured during play-back by counting the number of seconds a mouse rubbed its face and/or flinched its head during the session. The mice did not have access to food or water during the brief testing period. Behavioral evaluation was performed by an investigator blinded to the mouse group assignment. The behavior was characterized in 3 minute increments over the 60 minutes of evaluation. These data were entered into FileMaker Pro V7 (FileMaker Inc., Santa Clara CA) and exported to Excel (Microsoft Inc.) for analysis. Motor performance was assessed weekly using a Rotarod appliance (Columbus Instruments; Columbus OH) and measured as the ability of the mice to maintain balance on a rotating cylinder (20 rpm) by measuring the latency of each animal until it fell off.

### Statistical analysis

Data were compared by one way analysis of variance (ANOVA) followed by Tukey's post hoc test to determine differences between groups. *P *values less than 0.05 were considered statistically significant.

## Results

**Figure 1:**
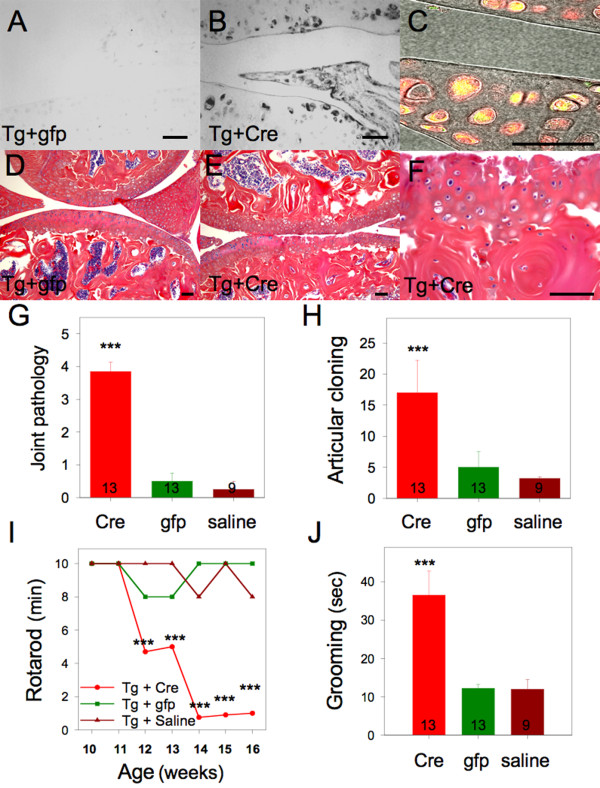
**Intra-articular IL-1β over-expression in the adult Col1-IL1β^XAT ^transgenic mouse results in joint pathology with behavioral changes**. (**A**) Intra-articular injection of FIV(gfp) in Col1-IL1β^XAT ^transgenic (**Tg**) mice (10 μL containing a total of 10^6 ^infectious particles) had no effect on IL-1β expression in the joints. In contrast, (**B**) intra-articular injection of FIV(**Cre**) in age matched transgenic mice (10 μL containing a total of 10^6 ^infectious particles) induced the expression of human IL-1β as detected by immunohistochemistry employing an antibody raised against the mature form of human IL-1β. Moreover, (**C**) cells infected by FIV(Cre) vector were detected by immunofluorescence (red) utilizing a Texas-Red conjugated antibody raised against the V5 epitope that tagged Cre recombinase in the FIV(Cre) vector (red fluorescence). The reporter gene β-galactosidase (the second ORF in the bicistronic Col1-IL1β^XAT ^transgene) was detected by a polyclonal antibody coupled to Alexa Fluor^® ^488 (green fluorescence). Therefore, cells infected by FIV(Cre) appear red and cells expressing β-galactosidase appear yellow due to the overlap of green+red. (**D**) Col1-IL1β^XAT ^transgenic (**Tg**) mice injected with the control vector FIV(**gfp**) (10 μL containing a total of 10^6 ^infectious particles) did not develop any articular pathology. (**E**) Conversely, Tg mice injected with FIV(Cre) intra-articularly developed joint pathology, (**F**) characterized by chondrocyte cloning, erosions and fibrillations. (**G**) Joint pathology was assessed on histology sections by a 0 - 5 scale. It was found that the mice that received FIV(Cre) intraarticularly (**Cre**) were characterized by a significant degree of joint pathology. (**H**) Articular cloning was employed as an additional measure of arthritis, whereby mice with intra-articular FIV(Cre) injection (**Cre**) were characterized by a significantly higher number of cloned chondrocytes in the articular cartilage. Furthermore, mice with arthritis displayed significantly decreased rotarod activity (**I**), employed here as a measure of joint dysfunction, as well as (**J**) significantly increased body grooming, as a measure of discomfort. *p < 0.05; **p < 0.01; ***p < 0.0001; Bar = 100 μM.

Intra-articular injection of the viral vector FIV(Cre) in the knees and TMJ's of Col1-IL1β^XAT ^transgenic mice induced the expression of human IL-1β following loxP directed excisional DNA recombination and transgene activation (Figure 1B-C). Eight weeks following viral transduction, we observed development of arthritis in experimental joints (knees), presenting as fibrillations and erosions of the articular cartilage (Figure 1E-F), whereas transgenic mice injected with the control vector FIV(gfp) showed no evidence of arthritis (Figure 1D). Joint pathology was assessed histologically (Figure 1G) on a scale 0-to-5 as well as on the number of chondrocyte clones in the articular cartilage (Figure 1H). Knee arthritis also induced behavioral changes including a decline in rotarod performance (Figure 1I) as well as increased grooming activity (Figure 1J). Joint pathology was also evaluated in APP/PS1 transgenic mice and was found to be indistinguishable from wild type mice. Histological evaluation of joints from APP/PS1; Col1-IL1β^XAT ^mice with osteoarthritis revealed joint pathology similar to that of Col1-IL1β^XAT ^mice with osteoarthritis (data not shown).

**Figure 2:**
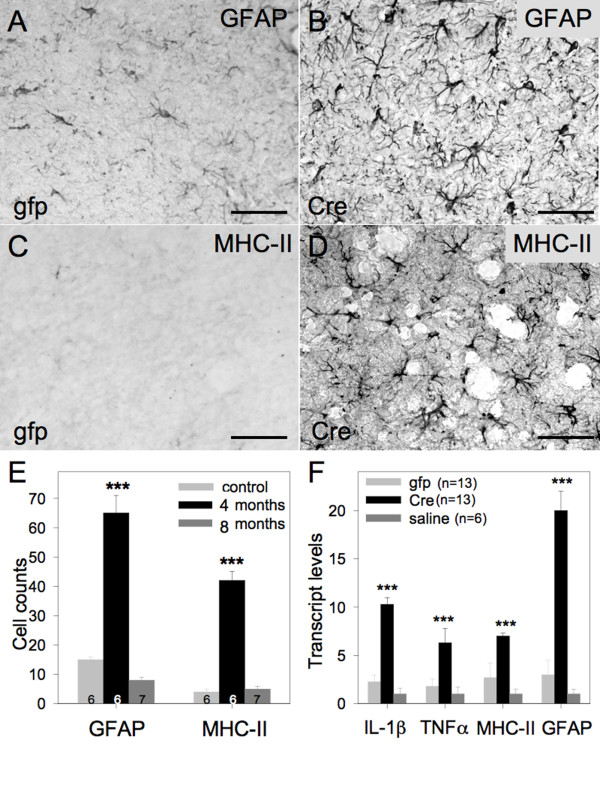
**Brain inflammation develops secondary to osteoarthritis**. (**A**) Col1-IL1β^XAT ^transgenic mice injected with FIV(gfp) in their joints presented baseline levels of GFAP expression. (**B**) Col1-IL1β^XAT ^transgenic mice injected with FIV(Cre) in their joints developed increased levels of GFAP expression as evaluated by immunohistochemistry. Similarly, (**C**) Col1-IL1β^XAT ^transgenic mice injected with FIV(gfp) lacked MHC-II staining in their brain, whereas (**D**) transgenic mice injected with FIV(Cre) in their joints displayed increased levels of MHC-II expression as evaluated by immunohistochemistry. The GFAP and MHC-II images were obtained from hypothalamic areas. (**E**) GFAP and MHC-II immunoreactive cells were counted at 2 and 6 months following FIV(gfp) (control) or FIV(Cre) injection in Col1-IL1β^XAT ^transgenic mice. A total of 19 mice was employed in this experiment. (**F**) Transcript levels for neuroinflammatory genes at 4 months of age were evaluated by real-time qRT-PCR in Col1-IL1β^XAT ^transgenic mice injected at 2 months of age with FIV(gfp), FIV(Cre) or saline. A total of 32 mice was employed in this study. ***p < 0.001; Bar = 50 μm. Mean ± SEM shown.

Two months following the induction of osteoarthritis, we observed astrocyte (Figure 2A-B) and microglia (Figure 2C-D) activation throughout the brains of affected mice. Although the number of reactive cells was significantly increased at 2 months, the level of glial cell activation normalized 6 months after osteoarthrtitis induction (Figure 2E), following the course of osteoarthritis development in this mouse model [23]. Consistent with these findings, real-time qRT-PCR analysis of inflammation-associated mRNAs in the mouse brain demonstrated a significant upregulation of murine IL-1β, TNFα, MHC-II and GFAP mRNA 2 months after the induction of osteoarthritis (Figure 2F), which returned to baseline levels at the 6 month time point.

We found no evidence of human IL-1β in the serum of any of the mice in the study as evaluated by ELISA. The absence of human IL-1β expression in the brain of the mice with osteoarthritis was confirmed by immunohistochemistry using an antibody raised specifically against a unique epitope of this cytokine that distinguishes it from murine IL-1β. Subsequently, we examined whether endogenous, murine cytokines were elevated in the blood stream of these mice: Mice injected with FIV(Cre) demonstrated a 3.8 fold increase (p < 0.016, F = 7.28) relative to controls (gfp injected) in serum levels of murine IL-6. Additional evidence for a systemic inflammatory response was revealed by immunohistochemistry in the livers of these mice, where we observed a dramatic increase in the number of MHC-II positive cells Kupfer cells and increased IL-6 expressing cells (data not shown).

**Figure 3:**
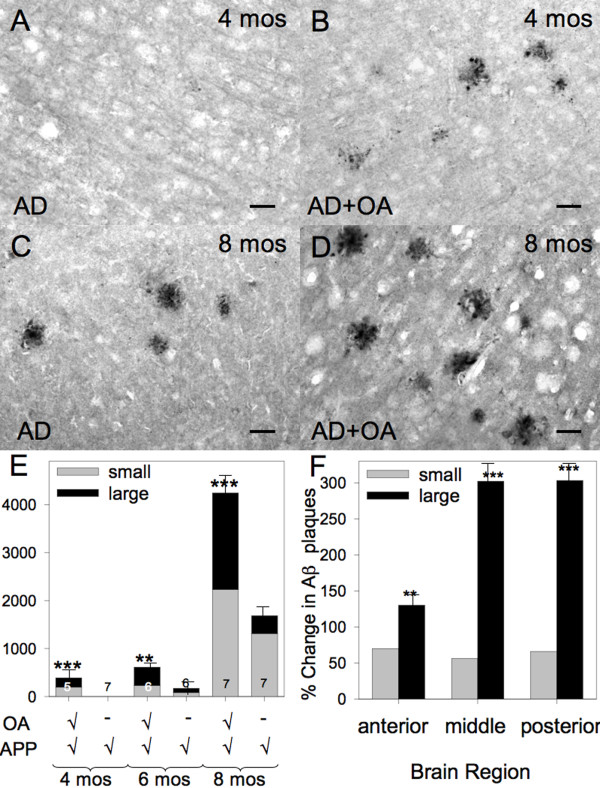
**Arthritis exacerbates and accelerates the development of Aβ plaques in mouse brain**. (**A**) Aβ plaques were not observed in the brain of 4 month old APP/PS1 transgenic mice. Conversely, (**B**) age and gender matched Col1-IL1β^XAT^;APP/PS1 mice with osteoarthritis presented Aβ-immunoreactive plaques scattered throughout the brain at 4 months of age. At 8 months of age, (**C**) APP/PS1 mice displayed Aβ plaque deposits throughout the brain parenchyma. (**D**) Age and gender matched Col1-IL1β^XAT^;APP/PS1 mice with osteoarthritis presented many more Aβ plaques. Overall, (**E**) APP/PS1 mice with arthritis displayed a significantly greater number of Aβ plaques at every time point examined (exacerbation effect), as well as developed Aβ plaque deposits the 4 month time point when no plaques were observed in APP/PS1 mice without arthritis (acceleration effect). (**F**) There was a modest increase in the number of small Aβ plaque deposits (< 100 μm) after osteoarthritis throughout the brain of Col1-IL1β^XAT^;APP/PS1 mice with osteoarthritis. The number of large Aβ plaques (> 100 μm), however, significantly increased in the mice with osteoarthritis, especially in the middle and posterior thirds of the brain. A total of 38 mice were included in this experiment: 20 Col1-IL1β^XAT^;APP/PS1 mice with osteoarthritis and 18 APP/PS1 mice without osteoarthritis. Mean ± SEM shown, ***p < 0.001; Bar = 100 μm.

**Figure 4:**
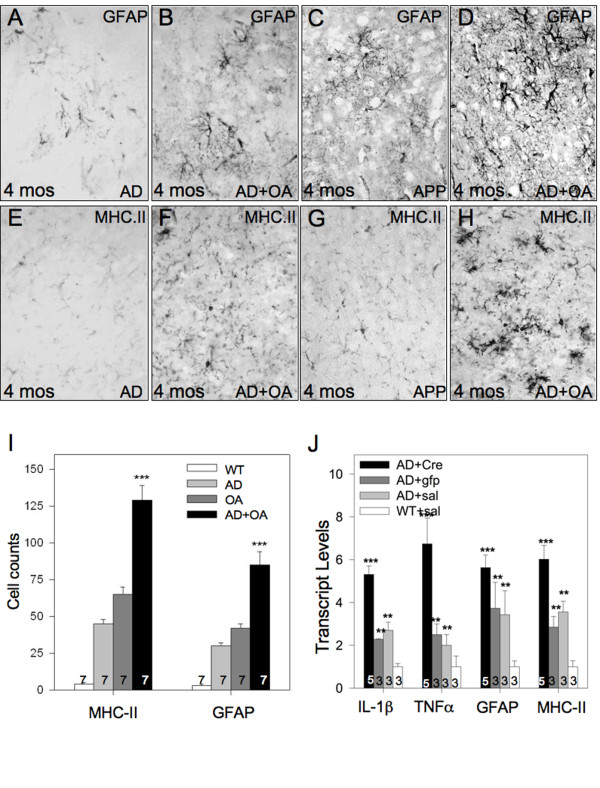
**Osteoarthritis exacerbates neuroinflammation in the presence of Aβ pathology**. **(A) **Four month old APP/PS1 transgenic mice displayed low numbers of GFAP positive astrocytes. (**B**) The induction of osteoarthritis in the APP/PS1 mouse model resulted in greater number of GFAP^+ ^astrocytes at the 4 month time point. (**C**) Eight month old APP/PS1 transgenic mice displayed low numbers of GFAP positive astrocytes, whereas (**D**) animals suffering from osteoarthritis presented with a greater number of reactive astrocytes as evaluated by GFAP immunohistochemistry. Moreover, (**E**) we observed only a few MHC-II positive cells in the brain of 4 month old APP/PS1 mice, whereas (**F**) a larger number was noted throughout the brain of Col1-IL1β^XAT^;APP/PS1 mice with osteoarthritis at the 4 month time point. (**G**) At eight months of age, we observed only a small number of MHC-II positive cells in APP/PS1 mice, whereas (**H**) a larger number was noted throughout the brain of Col1-IL1β^XAT^;APP/PS1 mice with osteoarthritis. (**I**) MHC-II and GFAP positive cells were quantified in the brains of 8 month wild type (**WT**), APP/PS1 (**AD**), Col1-IL1β^XAT ^mice with osteoarthritis (**OA**), and Col1-IL1β^XAT^;APP/PS1 mice with osteoarthritis (**AD+OA**). (**J**) Transcript levels for neuroinflammatory genes at 8 months of age were evaluated by real-time qRT-PCR in Col1-IL1β^XAT^;APP/PS1 mice injected with FIV(Cre), FIV(gfp), or saline, as well as wild type mice receiving saline (WT+sal). We observed an upregulation of glial cell activation in the Col1-IL1β^XAT^;APP/PS1 mice with osteoarthritis. Mean ± SEM shown, ***p < 0.001; Bar = 100 μm.

To determine whether the aforementioned osteoarthritis-induced neuroinflammation influences AD pathology, we induced osteoarthritis in APP/PS1; Col1-IL1β^XAT ^compound transgenic mice at 2 months of age. Activation of the Col1-IL1β^XAT ^transgene in this compound mouse model resulted in behavioral changes (reduction of locomotion) as assessed by the Rotarod method similar to those shown for Col1-IL1β^XAT ^mice with osteoarthritis (Figure 1I). Next, we identified the formation of Aβ plaques as early as the 4 months of age (2 month time point), we observed the development of Aβ plaque deposits in the brain parenchyma; conversely, there were no plaques observed in age- and gender- matched APP/PS1 mice (Figure 3A-B). At 8 months of age (6 month time point), APP/PS1; Col1-IL1β^XAT ^mice suffering from osteoarthritis displayed increased numbers of Aβ plaques throughout their brain compared to age- and gender- matched APP/PS1 mice, with an apparent preponderance of large diameter (> 100 μm) plaques (Figure 3C-D). To confirm these observations, we counted the number of large (> 100 μm) and small (< 100 μm) Aβ plaque deposits in APP/PS1 and APP/PS1; Col1-IL1β^XAT ^transgenic mice at 4, 6 and 8 months of age. We found that Aβ plaques appeared earlier in APP/PS1 mice with osteoarthritis, in significantly larger numbers at all time points examined (Figure 3E). When broken down by plaque size, we observed an approximately 50% increase in the number of small plaques throughout the brain parenchyma. In contrast, the increase of large plaques (> 100 μm) was much higher, especially in the middle and posterior third of the brain after arthritis induction (Figure 3F). Our results demonstrate that the presence of osteoarthritis, even in a small number of joints, induces the accumulation of Aβ plaques in the APP/PS1 model of AD at an age when such pathology is not present and enhances pathology at later times. Concomitant with the accelerated formation of Aβ plaque deposition in the APP/PS1; Col1-IL1β^XAT ^mice with osteoarthritis, we observed exacerbation of astrocyte (Figure 4A-D) and microglial (Figure 4E-H) activation as assessed by immunohistochemistry. The number of reactive glial cells was significantly increased in APP/PS1; Col1-IL1β^XAT ^mice with osteoarthritis compared to APP/PS1 mice without osteoarthritis and wild type controls (Figure 4I). mRNA analysis for several murine cytokines and markers of glial activation revealed increased transcript levels in the APP/PS1; Col1-IL1β^XAT ^mice with osteoarthritis compared to APP/PS1; Col1-IL1β^XAT ^mice without osteoarthritis or wild type controls (Figure 4J).

## Discussion

Our studies demonstrate that induction of osteoarthritis in the APP/PS1 mouse model of AD at 2 months of age resulted in the development of Aβ plaques and neuroinflammation as early as 4 months of age, whereas there was lack of Aβ plaques in the absence of osteoarthritis. APP/PS1 mice showed a modest level of Aβ pathology and neuroinflammation at 6 months of age, a time point when mice with osteoarthritis displayed a greater number of Aβ plaques. Aβ pathology and neuroinflammation was further exacerbated at the 8 month time point. These findings are consistent with the literature, whereby APP/PS1 transgenic mice begin developing Aβ plaque pathology at 5-6 months of age [26]. Overall, our data show that the induction of osteoarthritis in young adult APP/PS1;Col1-IL1**β**^XAT ^transgenic mice exacerbates and accelerates the development of AD pathology, suggesting that peripheral inflammation may be associated with increased risk for AD pathology.

Peripheral inflammation as a risk factor for AD was previously suggested by several clinical [1-6] and animal studies. For example, Cunningham and coworkers [9,10] examined the effects of acute systemic inflammation by means of LPS intraperitoneal injections in a mouse model of prion disease. They reported induction of acute behavioral and cognitive changes, along with acceleration of neurodegeneration and exacerbation of brain inflammation. Similar results were also reported by another study [27]. Intraperitoneal LPS injection in the PS1 transgenic mouse model of AD resulted in increased transcript levels for a number of inflammatory cytokines, such as IL-1β and TNFα, as well as induction in Aβ40 & Aβ42 levels in the brain [12]. In another study, LPS injection in the triple transgenic mouse model of AD (3xTg-AD) exacerbated Tau pathology by a cdk5 - mediated pathway, but did not have a measurable effect on Aβ [28]. Repeated LPS injections in wild type mice resulted in accumulation of Aβ1-42 in the hippocampus and cerebral cortex of mice through increased β- and γ-secretase activities along with increased expression of amyloid precursor protein [11].

Brain inflammation is considered an integral part of AD, sparked initially by observations of colocalization of MHC class II^+ ^microglia with neuritic plaques [29,30]. In the ensuing years, neuroinflammation was implicated as a primary contributor to AD pathogenesis based on epidemiologic studies linking chronic nonsteroidal anti-inflammatory drug (NSAID) use to reduced AD incidence [31] and the encouraging results of a few preliminary clinical studies (e.g. [30]). Subsequent clinical trials employing glucocorticoids [32] and NSAIDS [33,34] on patients with AD and mild cognitive impairment [35], as well as cognitively normal individuals at risk for AD [36], offered little support for the inflammatory hypothesis. Anti-inflammatory treatment of APP/PS1 double transgenic (2xTg-AD) mice had no effect on Aβ metabolism in the brain [37]. However, a subset of NSAIDS have been shown to possess γ-secretase modulating activity that can reduce Aβ production *in vitro *and *in vivo *independently of cyclooxygenase activity [38]. Previous studies in our laboratory, examining the role of brain inflammation in AD pathology, revealed that chronic, low level expression of IL-1β in the brain of GFAP-IL1β^XAT^; APP/PS1 compound transgenic mice resulted in amelioration of AD pathology via removal of Aβ plaques following the recruitment of peripheral immune cells in the brain [19,39].

But how is joint osteoarthritis linked to AD pathology? Numerous clinical and animal reports in the past showed an increase in circulating pro-inflammatory cytokines in the serum of patients and small animals suffering from arthritis [40]. To this end, our data showed a significant increase in IL-6 serum levels after the induction of osteoarthritis. A likely scenario is that circulating cytokines contribute to brain inflammation and may exacerbate it in the context of AD. There are several mechanisms by which cytokines might influence the CNS [41], including: (**A**) direct diffusion through the incomplete blood-brain barrier in the circumventricular organs; (**B**) activation of brain endothelial cells, which in turn signal to perivascular cells and cells of the brain parenchyma; (**C**) active transport of cytokines across the blood-brain barrier via transporter systems that can be shared between cytokines (IL-1α, IL-1β, IL1RA), or transporters for specific cytokines (TNFα); and (**D**) possible communication involving the vagus nerve or other neuronal afferents, which connect the peritoneal cavity with neuronal populations of the brain stem [41,42]. Although the exact mechanism by which circulating cytokines alter the CNS in our model is not known, it is anticipated that such signaling would result in exacerbation of the attendant glial cell activation and neuroinflammation in AD mice with osteoarthritis, which is exactly what our data demonstrate. It is interesting to note that neuroinflammation in our model of osteoarthritis was transient and resolved by the 6-month time point (8 months of age) in mice not carrying the APP/PS1 transgenes. In mice harboring such transgenes, pathology appears to continue to increase between 6 and 8 months, suggesting that a transient episode of peripheral inflammation is sufficient to trigger progressive AD pathology and neuroinflammation, perhaps through stimulation of a feed-forward process.

The specific mechanism linking peripherally induced neuroinflammation to AD pathology is not known, but might involve increased Aβ production [11,14,43], decreased Aβ catabolism, or changes in Aβ transport [44]. Alternatively, neuroinflammatory signals might limit the capacity of microglia and other cells to clear Aβ plaques [45]. Future studies focused on Aβ metabolism as well as investigation of inflammatory mediators and microglial phenotypes will be required in this model. A potentially fruitful study would be to compare the neuroinflammatory response in this model of peripheral inflammation where plaques accumulate to a model of CNS induced neuroinflammation where plaques are reduced (e.g. [39]).

Interestingly, physical exercise may reduce the degree of AD pathology in mice, raising the possibility that the changes we observed might be due to reduced locomotion in arthritic mice. Recent work on the subject reveals that short-term (1 month) locomotion exercise applied to AD mice (APP/PS1 and APP mutants) reduced total brain Aβ1-42 and Aβ1-40 levels, but did not influence plaque number [46,47]. However, long-term exercise (5 months) reportedly reduced Aβ plaque formation in the APP transgenic mouse [46]. Development of osteoarthritis in our model began to effect locomotion 2 weeks following transgene activation in the joints, imposing a potential impact on overall health for 6 weeks (short term effect). Notwithstanding differences in physical activity between normally caged mice and those undergoing experimentally induced exercise, these data together with the aforementioned studies suggest that loss of physical activity due to osteoarthritis likely has little or no effect on Aβ plaque loading evaluated in our studies.

In conclusion, the aforementioned body of literature as well as our own findings point out that peripheral inflammation exacerbates AD pathology in mice. These results have significant implications in consideration of risk factors for AD and possibly other neurodegenerative conditions. In particular, osteoarthritis is a very prevalent disease, with nearly 90% of individuals over the age of 65 having some degree of joint pathology. Future studies will focus on the mechanisms by which peripheral inflammation and blood borne cytokines contribute to increased AD pathology in our model. Strategies to reduce peripheral inflammation or that are aimed at the link between peripheral inflammation and the CNS may well prove beneficial in reducing the burden of neurodegenerative disease.

## Competing interests

The authors declare that they have no competing interests.

## Authors' contributions

SK contributed to the research design, research work and manuscript composition; RHT contributed to the research work and manuscript composition; JHM, MEO, RJ, MY, JAO and SMB contributed to the research work, and MKO contributed to the research design and manuscript composition. All authors read and approved the final manuscript.
